# Microscopic and Molecular Evaluation of Intestinal Microsporidia in Hemodialysis and Cancer Patients in Ilam Province

**DOI:** 10.1155/jotm/5072209

**Published:** 2026-08-17

**Authors:** Kosar Khezri, Nahid Maspi, Shahab Falahi, Hassan Nourmohammadi, Asad Mirzaei

**Affiliations:** ^1^ Department of Parasitology, School of Allied Medical Sciences, Ilam University of Medical Sciences, Ilam, Iran, medilam.ac.ir; ^2^ Zoonotic Diseases Research Center, Ilam University of Medical Sciences, Ilam, Iran, medilam.ac.ir; ^3^ Department of Hematology & Oncology, School of Medicine, Ilam University of Medical Sciences, Ilam, Iran, medilam.ac.ir

**Keywords:** cancer, *Enterocytozoon bieneusi*, genotyping, hemodialysis, immunodeficiency, Iran, microsporidia

## Abstract

**Background and Aims:**

Microsporidia are opportunistic intestinal pathogens causing chronic diarrhea in immunocompromised patients. Limited data exist on their prevalence in western Iran. This study aimed to investigate intestinal microsporidia in hemodialysis and cancer patients in Ilam Province.

**Methods:**

Stool samples from 78 immunocompromised patients (44 females, 34 males; mean age 53.5 ± 12.8 years) were examined microscopically after modified trichrome staining and molecularly by nested PCR targeting the ITS region. Phylogenetic analysis was performed using MEGA v10. Group comparisons were performed using the chi‐square or Fisher’s exact test, as appropriate.

**Results:**

Microscopy detected microsporidia in 10/78 samples (12.8%), while nested PCR identified 19/78 positive cases (24.4%; 95% CI: 16.2%–34.9%). *Enterocytozoon bieneusi* (*E. bieneusi*) was the predominant species (16/78; 20.5%), followed *by Encephalitozoon intestinalis* (2/78; 2.6%) and mixed infection (1/78; 1.3%). The prevalence was higher in hemodialysis patients (9/30; 30.0%) compared to cancer patients (10/48; 20.8%), but this difference was not statistically significant (*χ* ^2^ = 0.84, df = 1, *p* = 0.36). Due to the small sample size, this finding should be interpreted with caution and is presented as exploratory. No significant difference was observed by microscopy (Fisher’s exact test, *p* = 0.842). Phylogenetic analysis revealed the BEB6 genotype in all *E. bieneusi* isolates.

**Conclusion:**

This first report from Ilam Province demonstrates a notable prevalence of microsporidia, particularly *E. bieneusi* in hemodialysis and cancer patients. The BEB6 genotype raises the possibility of a zoonotic source, although no animal or environmental samples were analyzed to confirm transmission routes. While our findings are preliminary due to the limited sample size, they suggest that molecular diagnostic screening could be beneficial in similar high‐risk populations. However, larger multicenter studies are needed to confirm these findings before routine screening can be recommended.

## 1. Introduction

Microsporidia are obligate intracellular, spore‐forming eukaryotic pathogens capable of infecting a wide range of vertebrate and invertebrate hosts, including humans [1–3]. More than 1200 microsporidian species have been identified, most of which infect invertebrates and fish; however, at least 14 species belonging to eight genera have been reported in humans [4, 5]. Among these*, Enterocytozoon bieneusi* (*E. bieneusi*) and *Encephalitozoon* species, including *Encephalitozoon intestinalis (E. intestinalis)*, *Encephalitozoon cuniculi,* and *Encephalitozoon hellem,* are the most clinically relevant human pathogens [5, 6]. *E. bieneusi* and *E. intestinalis* are the predominant causes of intestinal microsporidiosis, with *E. bieneusi* accounting for more than 90% of reported intestinal infections [7].

Microsporidiosis is recognized as an important opportunistic infection, particularly among immunocompromised individuals such as patients with HIV/AIDS, cancer patients receiving chemotherapy, and those undergoing hemodialysis in these populations, impaired immune function increases susceptibility to infection and disease progression, leading to clinical manifestations ranging from chronic diarrhea and malabsorption to wasting syndrome and disseminated disease [1, 3, 6]. Hemodialysis and cancer patients were specifically chosen because they constitute the majority of immunocompromised patients in the study region and are at heightened risk for opportunistic enteric infections due to their sustained immunosuppressed status.

Microsporidia have been reported in 92 countries worldwide, with the highest prevalence rates observed in Northern Europe and South Africa [8]. The global prevalence of human microsporidiosis has been estimated at 9.2% [8], although considerably higher rates have been reported among immunocompromised populations. In Iran, the pooled prevalence of microsporidial infection among immunocompromised individuals is approximately 8.18%, increasing to 15.4% among patients with chronic diarrhea and 12.9% among those with CD4 counts below 200 cells/m [9]. Transmission occurs primarily through ingestion of environmentally resistant spores via contaminated water, food, or fecal–oral routes [4]. Poor sanitation, inadequate water quality, and zoonotic transmission have been identified as major factors facilitating the spread of microsporidia [10, 11].

Despite its clinical importance, microsporidiosis remains underdiagnosed. The small size of microsporidial spores and the limited ability of conventional microscopy to distinguish them from food particles, bacteria, and fungi often hinder accurate diagnosis [12]. In recent years, molecular techniques, particularly polymerase chain reaction (PCR)–based assays, have substantially improved the sensitivity and specificity of detection and have enabled reliable species identification, thereby enhancing epidemiological investigations and clinical diagnosis [13].

Although several studies have investigated human microsporidiosis in different regions of Iran, no data are available from Ilam Province in western Iran. This province borders Iraq and possesses environmental and socioeconomic characteristics that may favor the transmission of intestinal parasites, including microsporidia. Factors such as reliance on untreated water sources in some rural areas, agricultural practices involving animal manure, close human–animal contact, and active livestock movement across the border may increase the risk of exposure to microsporidial spores. However, the epidemiology of microsporidiosis in this region remains unknown. Therefore, the present study aimed to determine the prevalence of intestinal microsporidia among hemodialysis and cancer patients in Ilam Province using both microscopic and molecular methods. In addition, species identification was performed to provide baseline epidemiological data that may contribute to improved diagnostic, preventive, and control strategies for vulnerable populations in the region.

## 2. Materials and Methods

### 2.1. Study Population

A cross‐sectional study was conducted on 78 immunocompromised patients recruited from Danesh and Ebnesina Laboratories and the chemotherapy and hemodialysis units of Mostafa Khomeini Hospital in Ilam, Iran, between January 2022 and February 2023. The age range of participants was 25–82 years, and the gender distribution was 45% male and 55% female. All patients were residents of Ilam Province; the hospital does not routinely receive patients from other provinces. Sample size was determined using convenience sampling. All eligible patients who met the inclusion criteria and presented during the study period were invited to participate. Therefore, no a priori sample size or power calculation was performed.

### 2.2. Sample Collection

In this cross‐sectional descriptive study conducted in Ilam Province, 78 stool samples were collected from individuals with impaired immune systems, including 48 cancer patients and 30 hemodialysis cases. Before collecting the samples, participants completed the study‐specific form to obtain demographic data, including age, gender, residence, animal contact, education level, and current medical conditions (Supporting File S1). Samples were collected in sterile, leak‐proof containers and immediately transported to the Parasitology Laboratory at Ilam University of Medical Sciences for processing. A single stool sample was collected from each patient. Three smears were prepared from each stool sample for microscopic evaluation and fixed in absolute ethanolic alcohol. The remaining stool samples were aliquoted into sterile microtubes and stored at −20°C for subsequent molecular analysis.

### 2.3. Trichrome Staining and Microscopic Examination

Thin smears of each stool sample were prepared on glass slides and stained using the modified Ryan‐Blue trichrome method, optimized for microsporidia detection [14]. Fixed smears were immersed in the staining solution for 4 h at room temperature. After staining, slides were decolorized in 0.5% acid‐alcohol solution for less than 10 s. Dehydration was performed in three sequential steps. The slides were then cleared in xylene for 10 min. Stained slides were examined under a light microscope using a 100× oil immersion objective. All microscopic examinations were performed independently by two experienced parasitologists. In case of discrepancy, a third senior parasitologist was consulted, and the final result was reached by consensus. Microsporidia spores were identified based on their characteristic morphology and staining properties.

### 2.4. Molecular Identification

For molecular analysis, stool samples were thawed at room temperature. Approximately 1 g of each sample was suspended in 10 mL of distilled water, filtered through sterile gauze to remove debris, and washed with diethyl ether to remove lipids. The suspension was centrifuged at 7000 × g for 15 min, and the supernatant was discarded. The sediment was washed twice with phosphate‐buffered saline (PBS).

DNA extraction was carried out using a cetyltrimethylammonium bromide (CTAB)/chloroform‐isoamyl alcohol protocol, following the method described by Yazdanjooie. et al. [15]. Briefly, 300 µL of the processed sediment was transferred into a 2‐mL sterile microtube containing glass beads (0.45–0.52 mm diameter) and 200 µL of 10 mM Tris‐EDTA (TE) buffer. Then, 80 µL of sodium dodecyl sulfate (SDS) and 30 µL of proteinase K (20 mg/mL) were added. The tube was sealed with Parafilm and incubated at 60°C for 24 h with intermittent vortexing every 20 min. After incubation, the glass beads were removed, and 130 µL of NaCl was added, followed by 120 µL of CTAB‐NaCl solution. The mixture was incubated at 65°C for 10 min.

Next, 900 µL of chloroform‐isoamyl alcohol (24:1) was added, and the sample was centrifuged at 11,000 × g for 10 min. The aqueous phase (containing DNA) was transferred to a new 1.5‐mL microtube, and 0.6 volumes of ice‐cold isopropanol were added. After incubation at −20°C for 30 min, the sample was centrifuged at 12,000 × g for 15 min. The supernatant was discarded, and the pellet was washed with 1 mL of cold 70% ethanol, followed by centrifugation at 12,000 × g for 10 min. The supernatant was carefully removed, and the pellet was dried in a thermoblock. Finally, the DNA was dissolved in 50 µL of sterile distilled water.

The quality and quantity of the extracted DNA were assessed by 1% agarose gel electrophoresis and spectrophotometry (NanoDrop).

### 2.5. Nested PCR Amplification

Nested PCR was performed using species‐specific primers targeting the internal transcribed spacer (ITS) and small subunit ribosomal RNA (SSU‐rRNA) regions.

For *E. bieneusi*, a nested protocol adapted from Yang et al. [16] was used. The primary PCR employed outer primers EBITS3 (5′‐GGT CAT AGG GAT GAA GAG‐3′) and EBITS4 (5′‐TTC GAG TTC TTT CGC GCT C‐3′). The secondary PCR used inner primers EBITS1 (5′‐GCT CTG AAT ATC TAT GGC T‐3′) and EBITS2.4 (5′‐ATC GCC GAC GGA TCC AAG TG‐3′).

For *E. intestinalis*, a two‐step nested PCR targeting the SSU‐ITS and LSU regions of the rRNA gene was performed according to Katzwinkel‐Wladarsch et al. [17] and Valenčáková & Sučik [18]. Primary amplification used primers MSP1 (5′‐TGAATGKGTCCCTGT‐3′) and MSP2A (5′‐TCACTCGCCGCTACT‐3′). The nested reaction used inner primers MSP3 (5′‐GGAATTCACACCGCCCGTC(A/G) (C/T) TAT‐3′) and MSP4A (5′‐CCAAGCTTATGCTTAAGT(C/T) (A/C) AA(A/G) GGGT‐3′). Each 25 µL reaction contained 12.5 µL of 2× PCR master mix (Taq DNA polymerase, dNTPs, MgCl_2_), 1 µL each of forward and reverse primers (10 µM), 2 µL of template DNA, and 8.5 µL of nuclease‐free water. Positive controls for *E. bieneusi* and *E. intestinalis* were kindly provided by Dr. Hamed Mirjalali (Foodborne and Waterborne Diseases Research Center, Research Institute for Gastroenterology and Liver Diseases, Shahid Beheshti University of Medical Sciences, Tehran, Iran).

The thermal cycling conditions for both species are summarized in Table 1 (for the first and second rounds, identical conditions were applied for each species). PCR products were visualized on a 1.5% agarose gel stained with DNA‐safe dye.

**TABLE 1:** Thermal cycling conditions for nested PCR amplification of *Enterocytozoon bieneusi* (*E. bieneusi*) and *Encephalitozoon intestinalis (E. intestinalis)*.

Parameter	*E. bieneusi*	*E. intestinalis*
Initial denaturation	94°C, 5 min	95°C, 5 min
Denaturation	94°C, 30 s	95°C, 30 s
Annealing	57°C, 30 s	55°C, 45 s
Extension	72°C, 1 min	72°C, 1 min
Number of cycles	37	46
Final extension	72°C, 10 min	72°C, 10 min

*Note:* The same cycling conditions were used for both primary and secondary PCR for each species.

### 2.6. Phylogenetic Analysis

PCR products were purified using the FavorPrep Gel/PCR purification mini kit (Favorgen, Taiwan) according to the manufacturer’s instructions. Purified amplicons were sequenced using the Sanger method on an ABI 3500 DNA analyzer. The obtained ITS sequences were aligned using the MUSCLE algorithm implemented in MEGA X. The alignment was manually inspected and refined to remove ambiguous positions before phylogenetic reconstruction. The alignment was subsequently manually inspected and refined to eliminate ambiguous positions and optimize sequence homology. Phylogenetic analysis was performed using the Maximum Likelihood (ML) method with 1,000 bootstrap replicates. Bootstra *p* values ≥ 50% are shown on the tree. *E. intestinalis* (MG551530.1) was used as the outgroup [19]. All 19 PCR‐positive samples were sequenced. Of these, 7 *E. bieneusi* isolates with the highest sequence quality, representing both patient groups, were selected for phylogenetic tree construction. These sequences were deposited in GenBank under accession numbers PZ539175–PZ539181.

### 2.7. Statistical Analysis

Statistical analyses were performed using SPSS Version 26. Normality of age was assessed using the Shapiro–Wilk test. Normally distributed data are presented as mean ± SD; nonnormally distributed data as median (IQR). Proportions are reported with 95% confidence intervals (Wilson score method). For group comparisons of categorical variables, the Pearson chi‐square test was used when all expected cell counts were ≥ 5. When any expected cell count was < 5, Fisher’s exact test was applied instead. All reported *p* values are two‐sided. A *p* value < 0.05 was considered statistically significant.

## 3. Results

### 3.1. Demographic Characteristics of the Study Population

This study examined 78 immunocompromised patients (44 females, 34 males) residing in various cities within Ilam Province. The mean age of the participants was 53.5 ± 12.8 years (median: 54.0 years; IQR: 44.0–63.5 years; range: 25–82 years), and the majority (35.9%) were over 60 years old. Regarding education, 37.5% of participants were illiterate, while 20.0% held a university degree. Housewives (36.3%) and retirees (26.9%) were the most common occupational groups (Table 2).

**TABLE 2:** Demographic characteristics and microsporidia infection rates among participants.

Variable	Category	Abundance	Percentage	Positive cases	Infection rate (%)	*p* value
Gender	Female	44	55	7	15.9	0.052^†^
	Male	34	43.58	12	35.29	

Age (years)	< 30	1	1.28	0	0	0.098^‡^
	30–40	13	16.66	3	23.07	
	40–50	15	19.23	1	6.66	
	50–60	21	26.92	4	19.04	
	> 60	28	35.89	11	39.28	

Occupation	Housewife	29	36.25	7	24.13	0.568^†^
	Unemployed	12	15	3	25	
	Employee	5	6.25	0	0	
	Self‐employed	11	14.10	3	27.27	
	Retired	21	26.25	6	28.57	

Education	Illiterate	30	37.5	7	23.33	0.115^†^
	Elementary	6	7.5	3	50	
	Middle school	9	11.25	2	22.22	
	Diploma	11	13.75	2	18.18	
	University	16	20	5	31.25	

Underlying diseases	Cancer	48	60	10	20.83	0.36^†^
	Hemodialysis	30	37.5	9	30	

Animal contact	Yes	17	21.25	2	11.76	0.224^§^
	No	63	78.75	17	26.98	

*Note:* Percentages may not sum to 100% due to rounding. 95% confidence intervals (Wilson score method) are shown for infection rates. *p* values < 0.05 are considered statistically significant (none in this table).

^†^Pearson chi‐square test.

^‡^Chi‐square test for overall association (5 × 2 table).

^§^Fisher’s exact test (two‐sided).

### 3.2. Demographic Comparisons of Infection Rates

Comparison of infection rates across demographic subgroups revealed that age > 60 years was significantly associated with microsporidia infection (*χ* ^2^ = 5.54, df = 1, *p* = 0.019). The infection rate in patients over 60 years was 39.3% (11/28; 95% CI: 23.5%–57.6%) compared to 16.0% (8/50; 95% CI: 8.3%–28.6%) in patients aged ≤ 60 years.

Male patients had a higher infection rate (35.3%; 12/34; 95% CI: 21.2%–52.4%) compared to females (15.9%; 7/44; 95% CI: 7.9%–29.3%), although this difference did not reach statistical significance (*χ* ^2^ = 3.79, df = 1, *p* = 0.052).

No significant associations were found between infection and animal contact (2/17, 11.8% vs. 17/61, 27.9%; Fisher’s exact test, *p* = 0.224), education level (university: 5/16, 31.3% vs. nonuniversity: 14/62, 22.6%; Fisher’s exact test, *p* = 0.472), or occupation (*χ* ^2^ = 2.94, df = 4, *p* = 0.568). Other demographic variables listed in Table 2 (including residence) also showed no significant associations (data not shown).

### 3.3. Macroscopic Stool Assessment

Stool consistency was recorded for all participants. Diarrhea was observed in five patients (6.4%), all of whom were from the hemodialysis group and tested positive for microsporidia by nested PCR. Among the remaining 73 participants (93.6%) with normal stool consistency, 14 (19.2%) were positive for microsporidia.

### 3.4. Microscopic Stool Assessment

**FIGURE 1:**
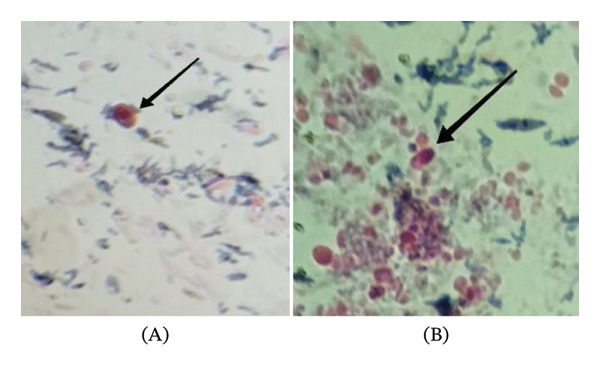
Representative microsporidial spores observed in human fecal smears stained with the modified trichrome (Ryan Blue) stain. Two independent positive specimens are shown (A, B). Arrows indicate oval to ovoid spores staining bright pink to red against a blue–green background. Microscopic examination was performed under oil immersion using a 100× objective (total magnification, 1000×).

Microsporidia spores were predominantly oval in shape and stained red‐pink with trichrome stain. Some spores exhibited a terminal vacuole or a central belt (Figures 1A, B). Of the 78 stool samples examined, 10 (12.8%; 95% CI: 7.1%–22.0%) were positive for microsporidia by light microscopy. The prevalence detected by microscopy was 12.5% (6/48; 95% CI: 5.8%–24.8%) among cancer patients and 13.3% (4/30; 95% CI: 5.1%–30.1%) among hemodialysis patients. No statistically significant difference was observed between the two groups (Fisher’s exact test, *p* = 0.842).

### 3.5. Molecular Detection

**FIGURE 2:**
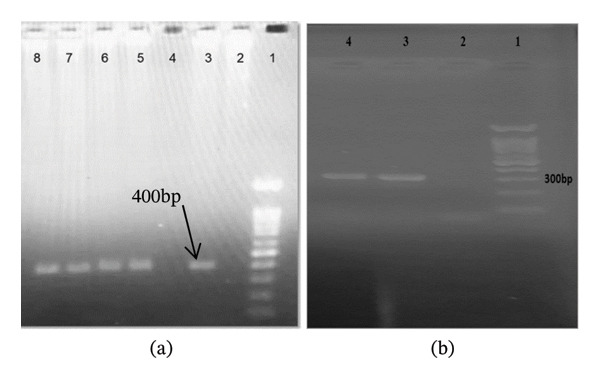
Analysis of nested PCR products by 1.5% agarose gel electrophoresis. (a) Amplification of the ITS region of *Enterocytozoon bieneusi* (approximately 400 bp). (b) Amplification of the ITS region of *Encephalitozoon intestinalis* (approximately 300 bp). In Panel A, Lane 1: 100‐bp DNA ladder; Lane 2: negative control; Lane 3: positive control (DNA kindly provided by Dr. H. Mirjalali); Lanes 5–8: clinical specimens positive for *E. bieneusi*. In Panel B, Lane 1: 100‐bp DNA ladder; Lane 2: negative control; Lane 3: positive control (same as in Panel A); Lane 4: clinical specimen positive for *E. intestinalis*. bp, base pairs.

Analysis of the nested PCR products by agarose gel electrophoresis demonstrated successful amplification of the ITS region, yielding an approximately 400‐bp amplicon specific for *E. bieneusi* (Figure 2a) and an approximately 300‐bp amplicon specific for *E. intestinalis* (Figure 2b).

The overall molecular prevalence of microsporidia infection was 24.4% (19/78; 95% CI: 16.2%–34.9%). Among cancer patients, the prevalence was 20.8% (10/48; 95% CI: 11.8%–34.3%), whereas among hemodialysis patients, it was 30.0% (9/30; 95% CI: 16.7%–47.9%). The difference between the two groups was not statistically significant (*χ* ^2^ = 0.84, df = 1, *p* = 0.36). Given the limited sample size, these findings should be interpreted with caution.

The agreement between light microscopy and nested PCR is summarized in Table 3. All 10 samples that tested positive by microscopy were also positive by nested PCR, whereas an additional nine samples were positive only by nested PCR. The overall agreement between the two methods was 88.5% (69/78), with a Cohen’s kappa coefficient of 0.63, indicating substantial agreement.

**TABLE 3:** Comparison of microscopic and nested PCR for detection of intestinal microsporidia (*n* = 78).

	PCR positive	PCR negative	Total^∗^
Microscopy positive	10	0	10
Microscopy negative	9	59	68
Total	19	59	78

*Note:* Cohen’s kappa=0.63 (substantial agreement).

^∗^Overall agreement=69/78 (88.5%).

Among the 19 PCR‐positive cases, 16 (20.5%; 95% CI: 13.1%–30.8%) were identified as *Enterocytozoon bieneusi*, two (2.6%; 95% CI: 0.7%–8.9%) as *Encephalitozoon intestinalis*, and one (1.3%; 95% CI: 0.2%–6.9%) as a mixed infection with both species. The mixed infection was detected in a cancer patient.

### 3.6. Phylogenetic Analysis

**FIGURE 3:**
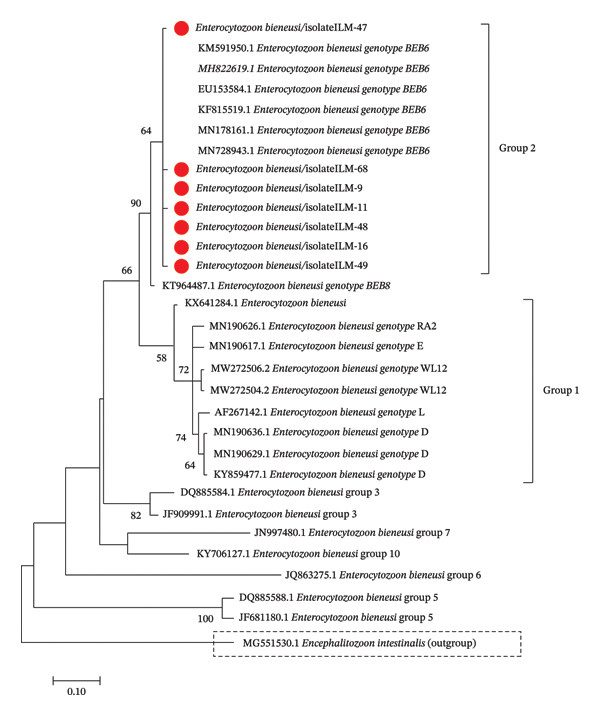
Maximum Likelihood (ML) phylogenetic tree based on the internal transcribed spacer (ITS) region sequences of *Enterocytozoon bieneusi*. The tree was inferred using the Maximum Likelihood method with 1,000 bootstrap replicates. Bootstrap support values (≥ 50%) are indicated at the corresponding nodes. *Encephalitozoon intestinalis* (GenBank accession no. MG551530.1) was used as the outgroup. The scale bar represents the number of nucleotide substitutions per site. Isolates sequenced in the present study (LM‐47, LM‐68, LM‐9, LM‐11, LM‐48, LM‐16, and LM‐49) are indicated by red filled circles (●).

(Figure 3) presents the phylogenetic tree based on the ITS region sequences of *Enterocytozoon bieneusi* isolates obtained in this study and reference sequences retrieved from GenBank, with *Encephalitozoon intestinalis* used as the outgroup. All 19 PCR‐positive samples were sequenced. Of these, seven representative *E. bieneusi* isolates (ILM‐9, ILM‐11, ILM‐16, ILM‐47, ILM‐48, ILM‐49, and ILM‐68) with the highest sequence quality were selected for phylogenetic tree construction. The remaining 12 sequences (3 *E. intestinalis* and 9 *E. bieneusi* with minor ambiguities) were excluded from the final tree.

All selected isolates clustered within Group 2 and were identified as the BEB6 genotype of *E. bieneusi*, forming a well‐supported clade with reference BEB6 sequences (e. g., GenBank accession nos. EU153584.1 and KMS91850.1), confirming their close genetic relationship. The sequences generated in this study have been deposited in GenBank under accession numbers PZ539175–PZ539181.

## 4. Discussion

Microsporidia are opportunistic pathogens causing chronic diarrhea in immunocompromised individuals. Global prevalence varies widely (0%–50%) [5]. Cancer patients receiving chemotherapy and hemodialysis patients exhibit heightened susceptibility to microbial infections due to sustained immunosuppression. In these populations, immune dysfunction is multifactorial: cancer patients may experience chemotherapy‐induced neutropenia and impaired cellular immunity, while hemodialysis patients often exhibit uremia‐associated immunosuppression, including defects in neutrophil and monocyte function, which increase their susceptibility to intestinal microsporidia [20].

Among demographic variables, only age > 60 years was significantly associated with infection (39.3% vs. 16.0%; *χ* ^2^ = 5.54, df = 1, *p* = 0.019). This finding aligns with immunosenescence, the age‐related decline in innate and adaptive immunity, and is consistent with Iranian studies in Kerman and Ahvaz that reported higher microsporidia prevalence in older immunocompromised patients [20, 21].

Male sex showed a higher infection rate (35.3% vs. 15.9%), but the difference was not statistically significant (*p* = 0.052). The near‐significant trend may reflect behavioral or occupational factors (e. g., men in Ilam more often work in agriculture or animal husbandry), but small sample size limits definitive conclusions.

No significant associations were found for occupation, education, or animal contact. Notably, participants reporting direct animal contact had a lower infection rate (11.8%) than those without (27.9%). This counter‐intuitive finding argues against direct household zoonotic transmission and instead supports an indirect environmental pathway (contamination of water, soil, or food via livestock manure). Similar patterns have been reported in rural areas where untreated manure is used as fertilizer and microsporidia spores have been detected in irrigation water and vegetables. Therefore, even individuals without direct animal contact may be exposed through contaminated produce or drinking water.

Nested PCR detected a significantly higher prevalence (24.4%) than microscopy (12.8%), consistent with the superior sensitivity of molecular methods. All 10 microscopies‐positive samples were also PCR‐positive, and nine additional samples were PCR‐positive only Table 3. The overall agreement was 88.5% (Cohen’s *κ* = 0.63), indicating substantial concordance.

The molecular prevalence in our study (24.4%) is comparable to reports from Alborz and Zahedan (21%–25%) but lower than in Ahvaz (36%) [21–23], possibly due to regional differences in sanitation, water quality, and climate. This rate is also higher than the 10% prevalence reported in cancer patients from Mazandaran Province [24] and exceeds findings from other Iranian studies (e. g., 15.3% in Shiraz [20]), but similar to others. The 30% prevalence in hemodialysis patients is noteworthy, though direct comparison with Egyptian data [25] is limited by different populations and settings.

A higher prevalence was observed in hemodialysis patients compared to cancer patients (30.0% vs. 20.8%; *p* = 0.36), but this difference was not statistically significant. Due to the small sample size and limited statistical power, this finding should be interpreted with great caution and is presented as exploratory only. No causal relationship can be inferred from this observation.

*E. bieneusi* predominated among positive cases, aligning with findings from multiple regions where it is the most common microsporidian species in high‐risk patient populations [20, 21, 26–28]. All *E. bieneusi* isolates belonged to the BEB6 genotype, commonly reported in livestock (e. g., cattle). Ilam Province has extensive agriculture with widespread use of untreated animal manure as fertilizer, which could hypothetically contaminate food and water sources. However, our data showed a lower infection rate among participants with direct animal contact (11.8%) than those without (27.9%). While this observation is intriguing, it does not confirm zoonotic transmission, as no animal or environmental samples were collected. If transmission does occur, it may be indirect (e.g., through contaminated water, soil, or food) rather than through direct household contact. Future environmental and molecular studies are needed to test this hypothesis and trace the exact transmission pathways. While our findings are exploratory, they suggest that clinicians might consider molecular screening for microsporidia in elderly immunocompromised patients, especially those with unexplained diarrhea. However, these recommendations are preliminary and require validation in larger, adequately powered studies before implementation in routine clinical practice.

This study has several limitations, including the use of convenience sampling, which may limit the generalizability of the findings; the cross‐sectional design, which precludes causal inference; and the relatively small sample size, which may have reduced the statistical power to detect differences between groups. Multivariable regression analyses were not performed due to the limited number of positive cases (*n* = 19), which would have compromised the reliability of adjusted estimates. In addition, no healthy control group was included, and detailed clinical data (e.g., cancer stage, chemotherapy regimen, duration of hemodialysis, and HIV status) were unavailable. Gastrointestinal symptom data were incomplete, with diarrhea documented in only five patients. Furthermore, animal and environmental samples were not collected; therefore, the potential transmission routes of *E. bieneusi* remain speculative.

## 5. Conclusion

In conclusion, this first report from Ilam Province reveals a notable prevalence (24.4%) of intestinal microsporidia, predominantly *E. bieneusi* (BEB6 genotype), in hemodialysis and cancer patients. Age > 60 years was identified as a significant risk factor. No difference in prevalence was observed between the two patient groups, although this finding is exploratory due to limited sample size. The presence of the BEB6 genotype, commonly associated with livestock, may suggest potential environmental or food‐borne transmission routes, but this hypothesis requires confirmation through future studies that include animal and environmental sampling*.* While our results are promising, the small sample size and exploratory nature of this study preclude strong clinical recommendations. Larger, multicenter studies with longitudinal designs are needed to confirm these findings and to assess the cost‐effectiveness of routine molecular screening for microsporidia in immunocompromised populations.

## Author Contributions

K.K. and N.M. made substantial contributions to the conception and design of the study. All authors contributed to experimental work and data acquisition, while K.K., N.M., and S.F. analyzed and interpreted the data. N.M. wrote the first draft of the manuscript and then critically revised it.

## Funding

The authors have nothing to report.

## Disclosure

All authors read and approved the final manuscript.

## Ethics Statement

This study was approved by the Ethics Committee of Ilam University of Medical Sciences, Ilam, Iran (Approval No: IR.MEDILAM.REC.1400.040), and conducted in accordance with the Declaration of Helsinki (1975, revised 2000). Informed consent was obtained from all study participants.

## Conflicts of Interest

The authors declare no conflicts of interest.

## Supporting Information

Additional supporting information can be found online in the Supporting Information section.

## Supporting information


**Supporting Information** File S1 contains the informed consent and demographic form used to obtain informed consent from all participants in the study, including the information sheet and the data collection tool for demographic and clinical characteristics.

## Data Availability

Data are available on request due to privacy/ethical restrictions.
