# Supplementary material for: Microscopic and Molecular Evaluation of Intestinal Microsporidia in Hemodialysis and Cancer Patients in Ilam Province
Source: J Trop Med. 2026 Aug 17;2026:5072209. doi: 10.1155/jotm/5072209 (PMC13482037; doi:10.1155/jotm/5072209)
Supplement: Supplementary file 1 [file JOTM-2026-5072209-s001.docx]

**Research Study Information and Consent Form**

**Study Title:** Microscopic and Molecular Evaluation of Intestinal Microsporidia in Hemodialysis and Cancer Patients in Ilam Province

**Principal Investigator:** Kosar Khezri, Master's Candidate in Medical Parasitology

**Institution:** Ilam University of Medical Sciences

**PART 1: Information Sheet**

**Introduction**

Dear Patient,

You are invited to participate in a research study as part of a Master's thesis in Medical Parasitology. Please take a moment to read this information carefully before deciding whether to participate.

**Purpose of the Study**

This study aims to investigate the presence of a specific intestinal parasite called microsporidia in patients receiving hemodialysis or undergoing cancer treatment in Ilam Province.

**What Will Happen?**

If you agree to participate, you will be asked to:

1. Complete a brief demographic questionnaire (about 5 minutes).

2. Submit a stool sample for laboratory testing.

**Potential Risks and Discomforts**

There are no expected physical risks other than minor discomfort from collecting a stool sample. Your information will be kept strictly confidential.

**Potential Benefits**

You may not receive any direct benefits; however, your participation could help improve understanding of infections in immunocompromised patients, potentially benefiting others in the future.

**Confidentiality**

Your information will be anonymized with a unique code instead of your name. Paper forms will be stored securely in a locked cabinet, and electronic data will be kept on a password-protected computer. Your identity will be protected in any published or presented results.

**Voluntary Participation**

Participation is completely voluntary. You may refuse or withdraw at any time without penalty or affecting your medical care.

**Contacts for Questions**

If you have any questions now or later, you can contact:

**• Researcher:** Kosar Khezri, khezrikosar548@gmail.com, +989183402629

**• Thesis Supervisor:** Nahid Maspi, nmaspi82@gmail.com, +989183458427

**PART 2: Informed Consent Agreement**

Please read each statement and initial in the boxes if you agree.

By initialing below, I confirm that:

 1. I have read and understood the information sheet and had the chance to ask questions.

2. I understand that my participation is voluntary and I can withdraw at any time without consequences.

3. I agree to provide a stool sample for microscopic and molecular analysis.

4. I agree to the use of my anonymized demographic and laboratory data for research.

**Participant's Name (Printed): ____________________________________**

**Participant's Signature: ____________________________________**

**Date: ______________________________**

**Researcher's Name (Printed): ____________________________________**

**Researcher's Signature: ____________________________________**

**Date: ______________________________**

**PART 3: Demographic Form**

All information is confidential and anonymized. Please check one box per question unless instructed otherwise.

Participant ID: ____________ (for office use only)

1. Date of Birth: ________________ OR Age: ____ years

2. Gender:

☐ Female

☐ Male

☐ Prefer not to say

3. Highest Education Level:

☐ Illiterate

☐ Primary School

☐ Secondary/High School

☐ Diploma

☐ Associate Degree

☐ Bachelor's Degree

☐ Postgraduate Degree

4. Employment Status:

☐ Employed

☐ Self-employed

☐ Unemployed

☐ Retired

☐ Homemaker/Housewife

5. Area of Residence:

☐ Urban

☐ Rural

6. Primary Medical Condition:

☐ Cancer (specify type if known: _______________)

☐ Renal Disease (on Hemodialysis)

☐ Organ Transplant

☐ Other Chronic Condition (please specify: ______________)

7. Contact with Animals (check all that apply):

☐ I have frequent contact with animals.

☐have no significant contact with animals.
